# Rice bran modulates renal disease risk factors in animals submitted to high sugar-fat diet

**DOI:** 10.1590/2175-8239-JBN-2020-0169

**Published:** 2021-01-15

**Authors:** Juliana Silva Siqueira, Fabiane Valentini Francisqueti-Ferron, Jéssica Leite Garcia, Carol Cristina Vágula de Almeida Silva, Mariane Róvero Costa, Erika Tiemi Nakandakare-Maia, Fernando Moreto, Ana Lúcia A. Ferreira, Igor Otávio Minatel, Artur Junio Togneri Ferron, Camila Renata Corrêa

**Affiliations:** 1 Universidade Estadual Paulista, Faculdade de Medicina, Botucatu, SP, Brasil.; 2 Universidade Estadual Paulista, Instituto de Biociências, Botucatu, SP, Brasil.

**Keywords:** Kidney Function Tests, Phytochemicals, Inflammation, Oxidative Stress, Testes de Função Renal, Compostos Fitoquímicos, Inflamação, Estresse Oxidativo

## Abstract

**Introduction:**

Obesity, diabetes, and hypertension are common risk factors for chronic kidney disease (CKD). CKD arises due to many pathological insults, including inflammation and oxidative stress, which affect renal function and destroy nephrons. Rice bran (RB) is rich in vitamins and minerals, and contains signiﬁcant amount of antioxidants. The aim of this study was to evaluate the preventive effect of RB on renal disease risk factors.

**Methods:**

Male Wistar rats (±325 g) were divided into two experimental groups to received a high sugar-fat diet (HSF, n = 8) or high sugar-fat diet with rice bran (HSF + RB, n = 8) for 20 weeks. At the end, renal function, body composition, metabolic parameters, renal inflammatory and oxidative stress markers were analyzed.

**Results:**

RB prevented obesity [AI (HSF= 9.92 ± 1.19 vs HSF + RB= 6.62 ± 0.78)], insulin resistance [HOMA (HSF= 83 ± 8 *vs*. HSF + RB= 42 ± 11)], dyslipidemia [TG (HSF= 167 ± 41 *vs*. HSF + RB=92 ± 40)], inflammation [TNF-α (HSF= 80 ± 12 *vs*. HSF + RB=57 ± 14), IL-6 (903 ± 274 *vs*. HSF + RB=535 ± 277)], oxidative stress [protein carbonylation (HSF= 3.38 ± 0.18 *vs*. HSF + RB=2.68 ± 0.29), RAGE (HSF=702 ± 36 *vs*. RSF + RB=570 ± 190)], and renal disease [protein/creatinine ratio (HSF=1.10 ± 0.38 *vs*. HSF + RB=0.49 ± 0.16)].

**Conclusion:**

In conclusion, rice bran prevented renal disease by modulating risk factors.

## Resumo

**Introdução:**

Obesidade, diabetes e hipertensão arterial são fatores de risco comuns para doenças renais crônicas (DRC). A DRC surge devido a muitos insultos patológicos, incluindo inflamação e estresse oxidativo, que afetam a função renal e destroem os néfrons. O farelo de arroz (FA) é rico em vitaminas e minerais, e contém uma quantidade significativa de antioxidantes. O objetivo deste estudo foi avaliar o efeito preventivo do FA nos fatores de risco de doenças renais.

**Métodos:**

Ratos Wistar machos (±325 g) foram divididos em dois grupos experimentais para receber uma dieta rica em gordura e açúcar (DRGA, n = 8) ou uma dieta rica em gordura e açúcar com farelo de arroz (DRGA + FA, n = 8) por 20 semanas. Ao final, foram analisados a função renal, composição corporal, parâmetros metabólicos, marcadores renais inflamatórios e de estresse oxidativo.

**Resultados:**

FA preveniu a obesidade [IA (DRGA= 9,92 ± 1,19 *vs*. DRGA + FA= 6,62 ± 0. 78)], resistência à insulina [HOMA (DRGA= 83 ± 8 vs DRGA + FA= 42 ± 11)], dislipidemia [TG (DRGA= 167 ± 41 *vs*. DRGA + FA=92 ± 40)], inflamação [FNT-α (DRGA= 80 ± 12 *vs*. DRGA + FA=57 ± 14), IL-6 (903 ± 274 *vs*. DRGA + FA= 535 ± 277)], estresse oxidativo [carbonilação de proteína (DRGA= 3. 38 ± 0,18 *vs*. DRGA + FA=2,68 ± 0,29), RAGE (DRGA=702 ± 36 *vs*. DRGA + FA=570 ± 190)], e doença renal [relação proteína/creatinina (DRGA=1,10 ± 0,38 *vs*. DRGA + FA=0,49 ± 0,16)].

**Conclusão:**

Em conclusão, o farelo de arroz preveniu doenças renais através da modulação dos fatores de risco.

## Introduction

Chronic kidney disease (CKD) is defined as changes in the kidney function or structure for more than three months, independent of the cause, which affect the health of an individual^1^. Epidemiological data show that CKD affects 10-16% of adults in the world^2^, being considered a global health problem. CKD diagnosis is usually established by the glomerular filtration rate (GFR). However, the reference GFR range does not exclude renal disease, since renal disease leads to decrease of renal function. Within this context, the National Kidney Foundation recommends proteinuria analysis for early stage detection and GFR estimations for assessing the progression of kidney disease^3^.

Obesity, diabetes, and hypertension are common risk factors for CKD^4^. CKD arises due to many pathological insults, including inflammation and oxidative stress, which affect the renal function and destroy nephrons. The literature reports an association between renal impairment and different mediators of inflammation including interleukin-6 (IL-6) and tumor necrosis factor-α (TNF-α) suggesting that CKD is a low-grade inflammatory process^5^^,^ 6.

Oxidative stress can be considered an imbalance in the reactive oxygen species (ROS) production/degradation ratio. Excessive ROS levels can produce cellular damage by interacting with biomolecules (proteins, lipids, and nucleic acids) resulting in negative effects on tissue function and structure, including kidney. Studies show that increased oxidative stress markers, as malondialdehyde (MDA) and carbonylated protein are inversely correlated with kidney function^5^^,^ 7. As a result, the nephrons compensate the function of injured nephrons with hyperfiltration, leading to glomerular hypertension, proteinuria, and eventually loss of renal function overt time^1^.

Several mechanisms are associated with renal inflammation and oxidative stress. When activated, the receptor for advanced glycation end products (RAGE), a multi-ligand member of the immunoglobulin superfamily of cell surface receptors, leads to a signalling sequence with the activation of the nuclear factor kappa-B (NFκB) resulting in proinflammatory cytokines production, such as TNF-α, IL-6, and monocyte chemoattractant protein-1 (MCP-1)^8^. RAGE activation can also directly induce oxidative stress by activating nicotinamide adenine dinucleotide phosphate (NADPH)-oxidase (NOX), especially NOX-4. Thus, RAGE activation is an interface between oxidative stress and inflammation, which are pillars for development of several diseases, especially in organs that express these AGE receptors, as brain, heart, and kidneys^9^.

In this context, an interest has emerged on the role of functional foods to prevent some diseases. Rice bran is one of the most abundant products produced in the rice milling industry that is rich in vitamins, including vitamin E, thiamin, niacin, and minerals like aluminum, calcium, chlorine, iron, magnesium, manganese, phosphorus, potassium, sodium, and zinc. It also contains a significant amount of antioxidants such as tocopherols, tocotrienols, and oryzanol. Rice bran also has proteins of high nutritional value and it is a good source of both soluble and insoluble dietary fiber^10^. Thus, considering that the consumption of high sugar-fat diet can lead to obesity and kidney disease risk factors development and the lack of studies regarding the effect of rice bran on these physiopathological aspects, the aim of this study was to evaluate the effect of rice bran on the modulation of renal disease risk factors in animals submitted to high sugar-fat diet.

## Material and methods

### Animals and experimental protocol

In the present study, male Wistar rats (±325 g) from the Animal Center of Botucatu Medical School, Sao Paulo State University (UNESP, Botucatu, SP, Brazil), were divided into two experimental groups to receive high sugar-fat diet (HSF, n = 8) or high sugar-fat diet with rice bran (HSF + RB, n = 8) for 20 weeks. The diets and water were provided *ad libitum*. The diet composition has been described in our previous study^11^. All the animals were housed in an environmentally controlled room (22±3 ºC, 12 h light-dark cycle and relative humidity of 60±5%). All of the experiments were performed in accordance with the Canadian Council on Animal Care (CCAC)^12^ and the procedures were approved by the Animal Ethics Committee of Botucatu Medical School (1305/2019). In order to confirm the effects of high sugar-fat diet on renal risk factors development in the HSF group, male Wistar (n=8, ±325 g, and same age), fed a standard diet, were used as reference group (baseline control group). At the end of the experiment, the animals were euthanized by decapitation after anesthesia with thiopental (120 mg/kg, intraperitoneal injection) and all efforts were made to minimize suffering. Blood from fasted animals was collected in tubes containing EDTA and centrifuged at 3500 rpm and the plasma was collected for analysis. Fat deposits and kidneys were collected for analysis.

### Rice bran dose

Since rice bran contains antinutritionals components, such as lipases and trypsin inhibitors^10^, it was subjected to a stabilization process, which consisted of heating in an oven to 100ºC, for 4 minutes. After the stabilization process, it was mixed to the chow in a dose of 11% (w/w). The dose has been chosen based on previous studies^13^.

### Nutritional parameters

The nutritional parameters evaluated were: chow intake, water intake, and caloric intake. Caloric intake was determined by multiplying the energy value of each diet (g × Kcal) by the daily food consumption plus the calories from water (0.25 × 4 × mL consumed).

### Body composition

Body composition was evaluated considering the final body weight (FBW), and adiposity index (AI). After euthanasia, fat tissues (visceral (VAT), epididymal (EAT), and retroperitoneal (RAT)) were used to calculate the AI by the following formula:

AI=VAT+EAT+RAT/FBW×100

14.

### Metabolic analysis

After 8 h fasting, blood was collected and the plasma was used to measure the biochemical parameters. Glucose concentration was determined using a glucometer (Accu-Chek Performa, Roche Diagnostics Brazil Limited) and triglycerides were measured with an automatic enzymatic analyzer system (Chemistry Analyzer BS-200, Mindray Medical International Limited, Shenzhen, China). The insulin level was measured using the enzyme-linked immunosorbent assay (ELISA) method using commercial kits (EMDMillipore Corporation, Billerica, MA, USA). The homeostatic model of insulin resistance (HOMA-IR) was used as an insulin resistance index, calculated according to the formula: HOMA-IR = (fasting glucose (mmol/L) x fasting insulin (µU/mL))/22.5^15^.

### Systolic blood pressure

Systolic blood pressure (SBP) was assessed in conscious rats by the noninvasive tail-cuff method with a Narco Bio-Systems^®^ electrosphygmomanometer (International Biomedical, Austin, TX, USA). The animals were kept in a wooden box (50 × 40 cm) between 38 and 40°C for 4-5 minutes to stimulate arterial vasodilation16. After this procedure, a cuff with a pneumatic pulse sensor was attached to the tail of each animal. The cuff was inflated to 200 mmHg pressure and subsequently deflated. The blood pressure values were recorded on a Gould RS 3200 polygraph (Gould Instrumental Valley View, Ohio, USA). The average of three pressure readings was recorded for each animal.

### Renal inflammation

Renal tissue (±150 mg) was homogenized (ULTRA-TURRAX^®^ T 25 basic IKA^®^ Werke, Staufen, Germany) in 1.0 mL of phosphate-buffered saline (PBS) pH 7.4 in cold solution and centrifuged at 800 *g* at 4°C for 10 min. The supernatant (100 *µ*L) was used in the analysis. TNF-α and IL-6 levels were measured using the ELISA method with commercial kits from R&D System, Minneapolis, USA. The supernatant (100 *µ*L) was used for analysis, and the results were corrected by the protein amount.

### Renal malondialdehyde levels (mda)

MDA level was used to evaluate the lipid peroxidation. Briefly, 250 µL of epididymal adipose tissue supernatant was used plus 750 µL of 10% trichloroacetic acid for precipitation of proteins. Samples were centrifuged (3000 rpm, for 5 minutes; Eppendorf^®^ Centrifuge 5804-R, Hamburg, Germany) and the supernatant removed. Thiobarbituric acid (TBA) was added 0.67% in ratio (1:1) and the samples heated for 15 minutes at 100°C. MDA reacts with TBA in the 1:2 (MDA:TBA) ratio. After cooling, the reading at 535nm was performed on Spectra Max 190 microplate reader (Molecular Devices^®^, Sunnyvale, CA, USA). The MDA concentration was obtained by the molar extinction coefficient (1.56 x 10^5^ M^-1^·cm^-1^) and the absorbance of the samples and the final result reported in nmol/g protein^17^.

### Renal protein carbonylation

Carbonylated proteins were measured by an unspecific method that uses DNPH (2,4-dinitrophenylhydrazine derivatizing agent) and photometric detection of any modified protein by carbonylation. Carbonylated protein levels are reported in nmol of DNPH/mg of protein^18^.

### Rage levels

Renal tissue (±150 mg) was homogenized (ULTRA-TURRAX^®^ T 25 basic IKA^®^ Werke, Staufen, Germany) in 1.0 mL of phosphate-buffered saline (PBS) pH 7.4 in cold solution and centrifuged at 800 g at 4°C for 10 min. The supernatant (100 µL) was used in the analysis. RAGE levels were measured with ELISA method using commercial kits from R&D System, Minneapolis, USA. The results were corrected according to the protein amount.

### Renal function

At 24 hours, urine was collected from the metabolic cages to measure the excretion of creatinine and the total protein. All analyses were performed with an automatic enzymatic analyzer system (biochemical analyzer BS-200, Mindray, China). The glomerular filtration rate (GFR = (urine creatinine × flux)/plasma creatinine) and proteinuria (protein/creatinine ratio) were also calculated.

### Statistical analysis

The data were submitted to Kolmogorov-Smirnov normality test. Parametric variables were compared by *Student’s* *t*-test and the results are reported as mean ± standard deviation. Non-parametric variables were compared by Mann-Whitney test and the results are reported as median (interquartile range (25-75)). Pearson correlation was used to assess the association among parameters. Statistical analyses were performed using Sigma Stat for Windows Version 3.5 (Systat Software Inc., San Jose, CA, USA). A *p* value < 0.05 was considered statistically significant.

## Results

### Rice bran effect on nutritional parameters

**Figure 1.**
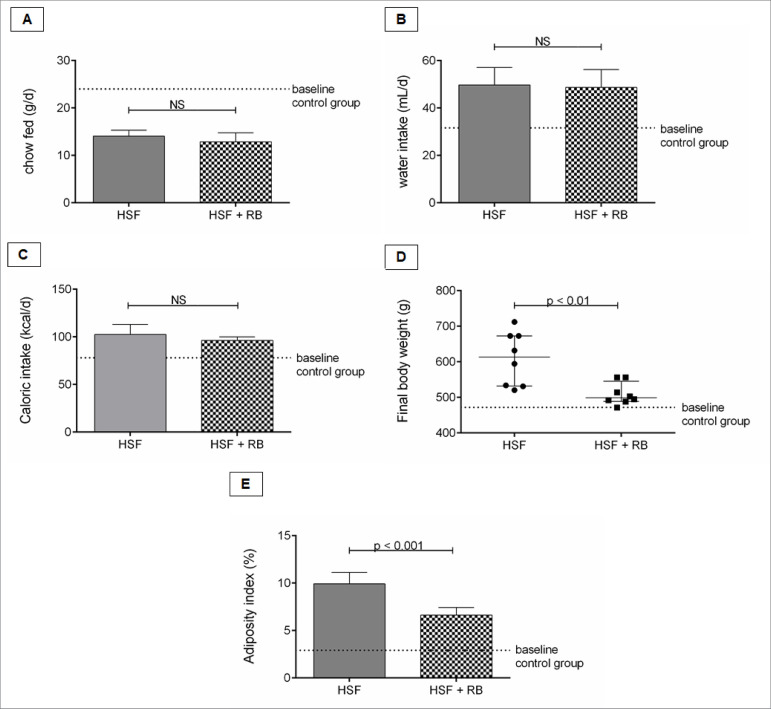
Nutritional parameters. A, Chow fed (g/day); B, Water intake (mL/day); C, Caloric intake (kcal/day); D, Final body weight (g); E, Adiposity index (%). Comparison by Student’s t-test or Mann-Whitney test, n=8 animals/group. p < 0.05 was considered significant. HSF: high sugar-fat diet; RB: rice bran. NS: not significant.

The nutritional parameters are presented in the Figure 1. It is possible to note the chow, water, and caloric intake in the HSF and HSF + RB groups. The HSF + RB showed lower final body weight and adiposity index than the HSF group.

### Rice bran effect on renal cardiometabolic risk factors

**Figure 2.**
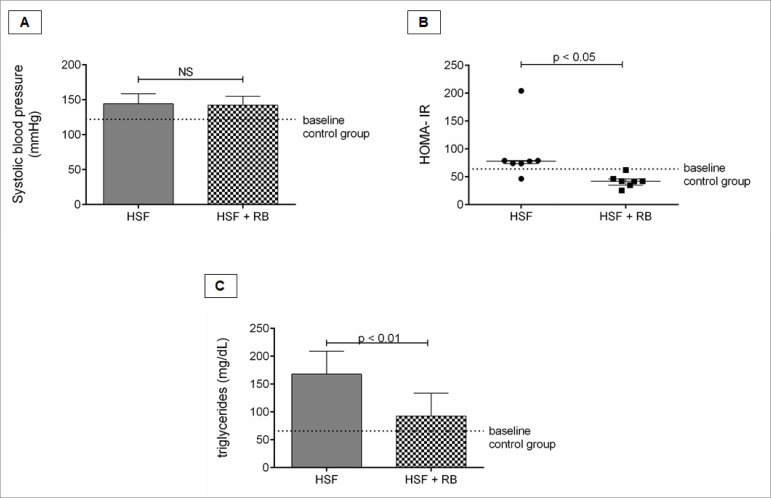
Renal cardiometabolic risk factors. A, Systolic blood pressure (mmHg); B, HOMA- IR; C, Triglycerides (mg/dL). Comparison by Student’s t-test or Mann-Whitney test, n=8 animals/group. p < 0.05 was considered significant. HSF: high sugar-fat diet; RB: rice bran. NS: not significant.

Renal cardiometabolic risk factors are presented in the Figure 2. It is possible to verify reduced HOMA-IR and triglycerides in the HSF + RB group compared to HSF. No rice bran effect was observed on systolic blood pressure.

### Rice bran effect on renal inflammation

**Figure 3.**
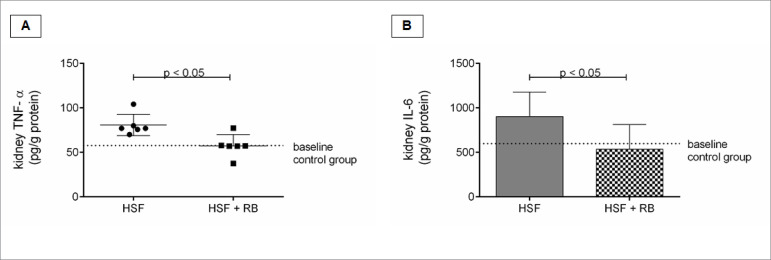
Renal inflammation parameters. A, Tumor necrosis factor alpha (TNF-α, pg/g protein); B, Interleukin-6 (IL-6, pg/g protein). Comparison by Student’s t-test or Mann-Whitney test. p < 0.05 was considered significant. NS: not significant.

Kidney inflammation parameters are presented in the Figure 3. Rice bran was effective to reduce inflammation, since the HSF + RB showed lower TNF-α and IL-6 levels compared to HSF.

### Rice bran effect on renal oxidative stress

**Figure 4.**
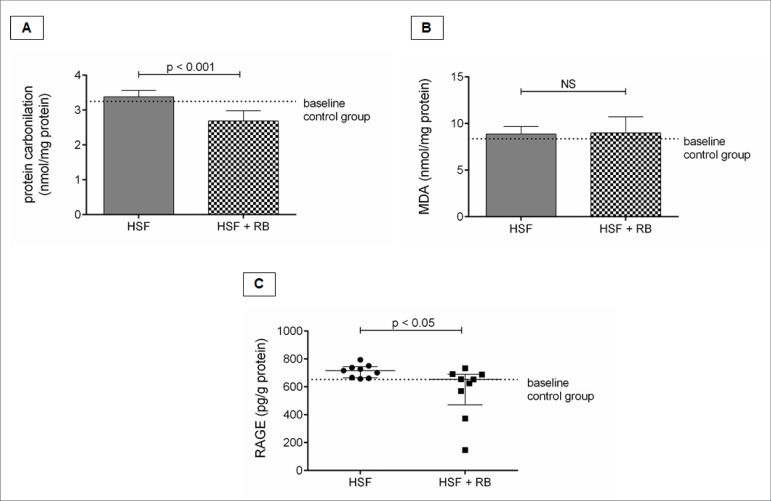
Renal oxidative stress parameters. A, Protein carbonylation (nmol/mg protein) B, Malondialdehyde (nmom/mg protein); C, RAGE (pg/g protein). Comparison by Student’s t-test or Mann-Whitney test. p < 0.05 was considered significant. NS: not significant.

Figure 4 shows the oxidative stress parameters. The group HSF + RB presented lower protein carbonylation and RAGE level compared to the HSF. No difference was observed for the MDA levels.

Malondialdehyde (nmom/mg protein); C, RAGE (pg/g protein). Comparison by Student’s t-test or Mann-Whitney test. *p* < 0.05 was considered significant. NS: not significant.

### Renal function parameters

**Figure 5.**
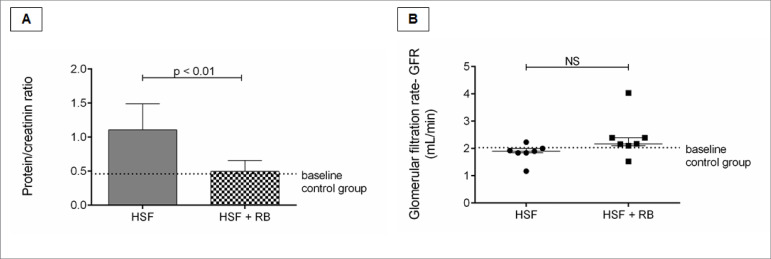
Renal function evaluation. A, Protein/creatinine ratio; B, Glomerular filtration rate (GFR, mL/min). Comparison by Student’s t-test or Mann-Whitney test. p < 0.05 was considered significant. NS: not significant.

Figure 5 presents the renal function parameters. It is possible to verify the proteinuria presence in the HSF group while the HSF + RB was protected. No difference was observed for the glomerular filtration rate between HSF and HSF + RB groups.

### Correlation among the parameters

**Figure 6.**
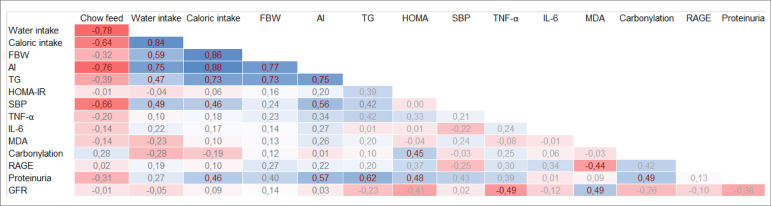
Pearson correlation among the variables. Red values indicate negative significant difference, gray values indicate non-significant difference, and blue values indicate positive significant correlation.

A positive correlation was found between proteinuria and caloric intake, adiposity index, triglycerides, HOMA, and carbonylation. Regarding the GFR, there was a positive correlation with MDA and a negative correlation with TNF-α (Figure 6).

## Discussion

The study aimed to evaluate the effect of rice bran on the modulation of renal damage risk factors. Kidney disease has a major effect on global health, both as a direct cause of morbidity and mortality and as an important risk factor for cardiovascular disease. Moreover, CKD is preventable and treatable and deserves greater attention in global health policy decision making^19^. Thus, the discovery of natural products, as rice bran, able to prevent this condition is extremely relevant. In the present study, a beneficial effect of rice bran was observed on the main renal disease risk factors. At the same time, the HSF group showed proteinuria and several risk factors for kidney injury, among them: obesity, dyslipidemia, insulin resistance, inflammation, and oxidative stress^4^.

The literature is scarce of studies with rice bran and CKD. One study published by our research group found that γOz, the main bioactive compound of rice bran, was effective to recover obesity-induced kidney disease after 10 weeks of treatment in Wistar rats^20^. Another experimental study by Al-Okbi et al.^21^ found that γ-oryzanol (γ-O) and rice bran oil/γ-O mixture (RBO/γ-O) had protective effects on cardiovascular diseases and cardiorenal syndrome, similar to Francisqueti et al.^22^, which found a protective effect of γOz on cardiorenal metabolic syndrome.

Obesity has been identified as one of the main cause of kidney disease since it is associated with hemodynamic, structural, and histopathological alterations in the kidney, as well as metabolic and biochemical alterations that predispose to kidney disease^23^^,^ 24. The animals that received rice bran presented the same chow, water, and caloric intake, however lower final body weight and adiposity index than the HSF group. Although the mechanism by which rice bran protects against obesity is not clarified, the literature confirms this antiobesogenic effect and attributes the benefits to dietary fiber, oligosaccharides, hemicelluloses, and non-starchy polysaccharides as well as some water-soluble phytochemicals present in the rice bran^25^.

Obesity is the main risk factor for the development of chronic diseases, such as type 2 diabetes and cardiovascular diseases, which increases the risk for CKD^26^. Hyperglycemia increases the non-enzymatic reaction of glucose and other glycating compounds derived both from glucose and from increased fatty acid oxidation, which generates advanced glycation end products in complication-prone cell types, including kidney cells^27^. The HSF group not only developed obesity but also insulin resistance. However, the animals that received rice bran did not present insulin resistance, which can be explained by the protection against obesity in the HSF + RB group. An excessive adipose tissue is associated with a chronic low-grade inflammation that may explain the development of the obesity-related pathologies, such as type 2 diabetes mellitus^28^^,^ 29.

Hypertension is a major risk factor for renal disease^30^. Multiple mechanisms are involved in determination of renal damage in hypertension, such as the renin-angiotensin-aldosterone system (RAAS), oxidative stress, endothelial dysfunction, and inflammation^31^. In the present study, no effect of rice bran was observed on systolic blood pressure. However, the HSF + RB group presented protection against kidney damage, which can be explained by the effect on inflammation and oxidative stress. The main bioactive compound in RB is gamma-oryzanol, which has demonstrated antioxidative and anti-inflammatory effects^25^^,^ 32 also in kidneys of obese animals^20^.

The upregulation of pro-inflammatory cytokines, as IL-6 and TNF-α, mediated by AGE/RAGE and NFκB, increase oxidative stress, which leads to local and systemic inflammation, glomerular and tubular lesions, and proteinuria. Among cytokines, TNF-α is known to cause direct cytotoxicity and apoptosis of renal cells^33^^,^ 34. Oxidized molecules reflect the damage mediated by oxidative stress in cells and tissues, and their measurement can be indicative of oxidative stress in a specific disease, as well as the potential efficacy of clinical treatments. Some of these modifications, as carbonylation, are irreversible and can lead to altered protein expression and activity, resulting in organ impairment^35^. Confirming the antioxidant and anti-inflammatory effect of rice bran, the HSF + RB animals showed reduced TNF-α, IL-6, RAGE, protein carbonylation, and proteinuria compared to the HSF group.

In summary, rice bran was able to prevent obesity, insulin resistance, dyslipidemia, inflammation, oxidative stress, and renal disease. These findings provide important information about the use of bioactive compounds as alternative therapeutics for preventing renal disease and associated risk factors. However, since the main limitation of this study was not evaluating the pathways involved in the positive effects of rice bran, more studies are necessary. Therefore, we concluded that rice bran was able to prevent renal disease by modulating risk factors.
